# Femoral Hernia Containing the Right Fallopian Tube: A Rare Finding

**DOI:** 10.1055/s-0039-1693055

**Published:** 2019-08

**Authors:** Duarte Viveiros, André Lázaro, Hélder Carvalho

**Affiliations:** 1 General Surgery Department, Coimbra Hospital and University Center, Coimbra, Portugal; 2 Coimbra University, Medicine Faculty, Coimbra, Portugal

**Keywords:** femoral hernia, fallopian tube, ovary, infertility, hérnia femoral, trompa de falópio, ovário, infertilidade

## Abstract

Femoral hernias comprise a small proportion of all groin hernias. They are more common in women and have a high rate of incarceration and strangulation, leading to emergency repair. A 61-year-old female patient was admitted to the emergency department complaining of a 2-day painful lump in the right groin, that had become more intense in the last 24 hours. Physical examination suggested the presence of a strangulated femoral hernia, and the patient underwent emergency surgical repair. Intraoperatively, the right fallopian tube was observed in the hernia sac. Since there were no signs of ischemia, the tube was reduced back into the pelvic cavity and the hernia was repaired. The postoperative period was uneventful, and the patient was discharged without complications, 3 days after surgery.

## Resumo

As hérnias femorais representam uma pequena fração de todas as hérnia da região inguinal. Elas são mais comuns entre as mulheres e estão associadas a elevadas taxas de complicações, como encarceramento e estrangulamento, com necessidade de cirurgia urgente. Uma paciente do sexo feminino, de 61 anos, recorreu ao serviço de emergência por quadro de dor e tumefação da região inguinal direita com 2 dias de evolução e agravamento nas últimas 24 horas. O exame objetivo sugeria a presença de uma hérnia femoral encarcerada, e a paciente foi submetida a cirurgia urgente. Intraoperatoriamente, confirmou-se o diagnóstico de hérnia femoral encarcerada, que continha a trompa de falópio direita no interior do saco herniário. Uma vez que a que a trompa não apresentava sinais de isquemia, o conteúdo da hérnia foi reduzido, e procedeu-se à sua reparação. O período pós-operatório decorreu sem intercorrências, e a paciente teve alta no 3° dia após a cirurgia.

## Introduction

Femoral hernias are relatively uncommon and account for ∼ 2% of all hernias and 2 to 8% of all groin hernias. They are mostly observed among adults (40–70 years), much more common in women than in men, and are frequently associated with incarceration and strangulation.^1^ ^2^ ^3^ Increased intra-abdominal pressure, which occurs in certain conditions, such as obesity, chronic cough, heavy exercise or lifting, and pregnancy, is usually implicated.^4^ ^5^ ^6^ Different contents in the hernia sac have been described in the literature, but exclusive herniation of the fallopian tube is extremely uncommon.^7^ ^8^ We present a rare case of a 61-year-old female with a femoral incarcerated hernia containing a fallopian tube that required emergency surgical intervention at our institution.

## Case Presentation

A 61-year-old female patient was admitted to the emergency department complaining of a 2-day painful lump in the right groin, which gradually became tender to palpation during the last 24 hours. She denied urinary symptoms, anorexia, nausea, or vomiting and had regular bowel function. She had no significant past medical or surgical history. On clinical examination, the patient was afebrile, her pulse rate was 83 bpm and blood pressure 132/86 mmHg. Physical examination revealed a 3 × 4 cm tender mass in the right groin, irreducible and non-pulsatile. Abdominal examination showed mild tenderness in the right iliac fossa. The leucocyte count was 7,400/μl (neutrophils: 50%), and C-reactive protein was 0.84 mg/dL. The patient underwent ultrasonography that showed “signs compatible with right femoral hernia, non-reducible, containing intestine, fat and fluid.” Abdominal and thorax X-rays were unremarkable.

## Treatment

**Fig. 1:**
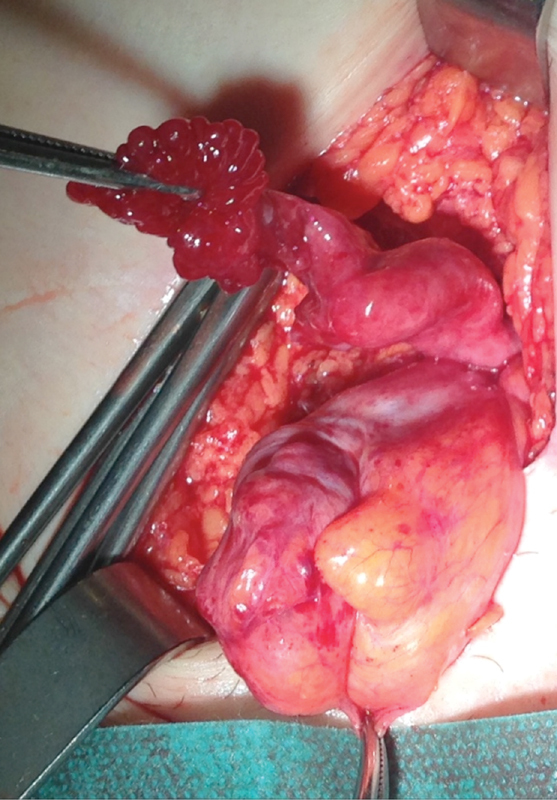
The uterine tube with its mesosalpinx, while the right ovary lies within the abdominal cavity.

**Fig. 2:**
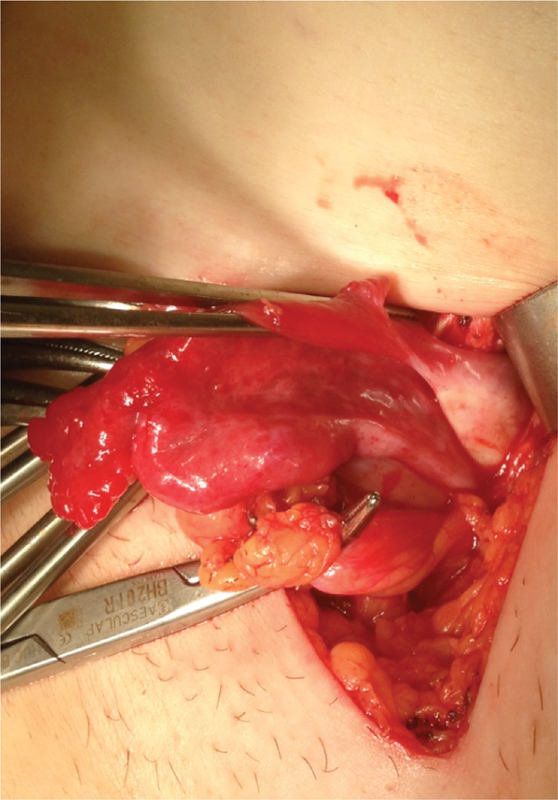
The uterine tube with its mesosalpinx, and the right ovary.

Clinical assessment suggested the presence of a strangulated femoral hernia, and the patient underwent emergency surgery. We used a lower inguinal approach and carefully exposed the femoral hernia sac. After opening the sac, we confirmed the diagnosis of femoral hernia that unexpectedly contained the right fallopian tube (Figs. 1 and 2). The tube was congested, but showed no signs of ischemia and was reintroduced into the pelvic cavity, without any difficulty. We excised the hernia's sac and repaired the defect using a polypropylene mesh plug.

## Outcome and Follow-Up

The postoperative period was uneventful, and the patient was discharged without complications 3 days after the surgery. No signs of hernia recurrence were noted at 1-, 3-, and 6-month follow-up appointments.

## Discussion

Femoral hernias comprise a small proportion of all groin hernias, accounting for ∼ 2 to 8% of cases.^2^ They are 4-to-5-fold more common in women, generally occur in the elderly, and are more frequent on the right side.^1^ ^2^ ^3^ ^4^ ^5^ ^6^ This specific type of hernia occurs when intraabdominal content protrudes through the femoral ring into the femoral canal, beneath the inguinal ligament. Because of their narrow neck and rigid ligamentous borders, they are prone to incarceration and strangulation, leading to emergency surgery in many cases.^9^ ^10^ Femoral hernias usually contain preperitoneal fat or segments of the small bowel; nonetheless, other contents, such as stomach, colon, appendix, bladder and Meckel diverticulum have been described in the literature. Herniation of the fallopian tube or ovary is an extremely rare condition, especially in adults, due to their normal anatomical position, located at a lower level than the femoral ring.^11^ Maylard^12^ described one of the first cases of a femoral hernia containing the ipsilateral fallopian tube, in 1892. Since then, a few of cases have been reported, and most of them were found in the pediatric population. Typical femoral hernias present as a tender, non-reducible groin lump, with no cough impulse, situated below and lateral to the pubic tubercle. The diagnosis is generally done by physical examination; however, in some patients, imaging exams, such as abdominal X-ray, ultrasonography, CT, or MRI, may be useful in the diagnosis, especially because of the variety of possible contents.^8^ ^11^ Preoperative diagnosis of a strangulated fallopian tube in a femoral hernia is extremely difficult, as only one case is described in the literature.^13^

Early diagnosis and surgical treatment are key factors for the prognosis, since female adnexa are particularly vulnerable to ischemia when entrapped, which may lead to an infarcted and unsalvageable ovary or fallopian tube. Therefore, female adnexa should always be considered as possible hernia contents to warrant a prompt assessment and intervention, thus avoiding the necessity of resection and preserving fertility in women of childbearing age.^11^ ^14^ ^15^ Surgical approach of a femoral hernia containing female adnexa follows the same principles of any other femoral hernia treatment. There are many different repair techniques that can be divided in two main groups: tension-free mesh techniques (open or laparoscopic), or non-mesh techniques. In our case, we have decided to do a tension-free repair using a polypropylene mesh plug, known to have lower recurrence rates and associated to less short-term pain and discomfort, allowing for a faster recovery and rapid return to normal activities.^1^

## References

[1] Hachisuka T Femoral hernia repair Surg Clin North Am 2003 83 05 1189 1205 14533910 10.1016/S0039-6109(03)00120-8

[2] Dahlstrand U Wollert S Nordin P Sandblom G Gunnarsson U Emergency femoral hernia repair: a study based on a national register Ann Surg 2009 249 04 672 676. Doi: 10.1097/SLA.0b013e31819ed943 19300219 10.1097/SLA.0b013e31819ed943

[3] Kark A E Kurzer M Groin hernias in women Hernia 2008 12 03 267 270. Doi: 10.1007/s10029-007-0330-4 18214638 10.1007/s10029-007-0330-4

[4] Keith A On the origin and nature of hernia Br J Surg 1923 11 455 475. Doi: 10.1002/bjs.1800114307

[5] McVay C B Savage L E Etiology of femoral hernia Ann Surg 1961 154 06 25 32. Doi: 10.1097/00000658-196112000-00005 17859685 10.1097/00000658-196112000-00005 PMC1466821

[6] Atmatzidis S Chatzimavroudis G Dragoumis D Atmatzidis K Incarcerated femoral hernia containing ipsilateral fallopian tube Case Rep Med 2010 2010 741915. Doi: 10.1155/2010/741915 20981264 10.1155/2010/741915 PMC2964038

[7] Smolentsev I A [Strangulation of a uterine tube in a femoral hernia] Vestn Khir Im I I Grek 1973 110 04 136 4712354

[8] Alzaraa A Unusual contents of the femoral hernia ISRN Obstet Gynecol 2011 2011 717924. Doi: 10.5402/2011/717924 21647228 10.5402/2011/717924 PMC3101789

[9] Heys S D Brittenden J Strangulated femoral hernia: the persisting clinical trap Postgrad Med J 1991 67 783 57 59. Doi: 10.1136/pgmj.67.783.57 2057430 10.1136/pgmj.67.783.57 PMC2398924

[10] Sorelli P G El-Masry N S Garrett W V Open femoral hernia repair: one skin incision for all World J Emerg Surg 2009 4 44. Doi: 10.1186/1749-7922-4-44 19948016 10.1186/1749-7922-4-44 PMC2789711

[11] Gurer A Ozdogan M Ozlem N Yildirim A Kulacoglu H Aydin R Uncommon content in groin hernia sac Hernia 2006 10 02 152 155. Doi: 10.1007/s10029-005-0036-4 16172801 10.1007/s10029-005-0036-4

[12] Maylard A E Strangulated hernia of the left ovary in the femoral region BMJ 1892 1(1632):761 762. Doi: 10.1136/bmj.1.1632.761 10.1136/bmj.1.1632.761 PMC2420081 20753636

[13] Oakenful C Lambrianides A L Incarcerated adult femoral hernia containing a fallopian tube Australas J Ultrasound Med 2011 14 04 16 17. Doi: 10.1002/j.2205-0140.2011.tb00125.x 28191125 10.1002/j.2205-0140.2011.tb00125.x PMC5024908

[14] Anders J F Powell E C Urgency of evaluation and outcome of acute ovarian torsion in pediatric patients Arch Pediatr Adolesc Med 2005 159 06 532 535. Doi: 10.1001/archpedi.159.6.532 15939851 10.1001/archpedi.159.6.532

[15] Yamashita Y Sowter M Ueki M Gudex G Adnexal torsion Aust N Z J Obstet Gynaecol 1999 39 02 174 177. Doi: 10.1111/j.1479-828X.1999.tb03365.x 10755772 10.1111/j.1479-828x.1999.tb03365.x

